# Hypothalamic CREB Regulates the Expression of Pomc-Processing Enzyme Pcsk2

**DOI:** 10.3390/cells11131996

**Published:** 2022-06-22

**Authors:** Ariane Maria Zanesco, Natalia F. Mendes, Daiane F. Engel, Rodrigo S. Gaspar, Davi Sidarta-Oliveira, José Donato, Licio A. Velloso

**Affiliations:** 1 School of Medicical Sciences, University of Campinas, Campinas 13083-887, Brazil; arianemariazanesco@gmail.com (A.M.Z.); natalia.mendesss@gmail.com (N.F.M.); daiane.engel01@gmail.com (D.F.E.); gaspar.rodrigo@live.com (R.S.G.); davisidarta@gmail.com (D.S.-O.); 2 Laboratory of Cell Signaling, Obesity and Comorbidities Research Center, University of Campinas, Campinas 13083-864, Brazil; 3 School of Pharmacy, Federal University of Ouro Preto, Ouro Preto 35400-000, Brazil; 4 Department of Physiology and Biophysics, University of São Paulo, São Paulo 05508-900, Brazil; jdonato@icb.usp.br

**Keywords:** proopiomelanocortin, obesity, diet, saturated fatty acid, CREB

## Abstract

Background: The hypothalamic proopiomelanocortin (Pomc) neurons act as first-order sensors of systemic energy stores, providing signals that regulate caloric intake and energy expenditure. In experimental obesity, dietary saturated fatty acids affect Pomc endopeptidases (PCs), resulting in the abnormal production of the neurotransmitters α-melanocyte-stimulating hormone (α-MSH) and β-endorphin, thus impacting energy balance. The cAMP response element-binding protein (CREB) is one of the transcription factors that control the expression of Pomc endopeptidases; however, it was previously unknown if dietary fats could affect CREB and consequently the expression of Pomc endopeptidases. Methods: Here, we used single-cell RNA sequencing analysis, PCR, immunoblot, ELISA and immunofluorescence histological assays to determine the impact of a high-fat diet (HFD) on the expression and function of hypothalamic CREB and its impact on the melanocortinergic system. Results: The results indicate that CREB is expressed in arcuate nucleus Pomc neurons and is activated as early as nine hours after the introduction of a high-fat diet. The inhibition of hypothalamic CREB using a short-hairpin RNA lentiviral vector resulted in increased diet-induced body-mass gain and reduced energy expenditure. This was accompanied by reduced expression of the Pomc endopeptidases, protein convertase 2, which are encoded by Pcsk2, and by the loss of the high-fat-diet-induced effect to inhibit the production of α-MSH. Conclusions: This study provides the first evidence for the involvement of CREB in the abnormal regulation of the hypothalamic Pomc endopeptidase system in experimental obesity.

## 1. Introduction

The hypothalamic melanocortin system is composed of a network of neurons that sense the energy stores in the body and respond by controlling caloric intake and energy expenditure, which, under physiological conditions, maintain body-mass stability [1,2]. Proopiomelanocortin (Pomc) neurons are a key component of this system; they are activated by leptin, insulin, glucagon-like peptide 1 (GLP1), and certain nutrients promoting satiety and increasing energy expenditure [3,4]. Experimental studies have shown that the excessive consumption of saturated fatty acids can damage Pomc neurons, thereby promoting body-mass gain [5].

**Figure 1:**
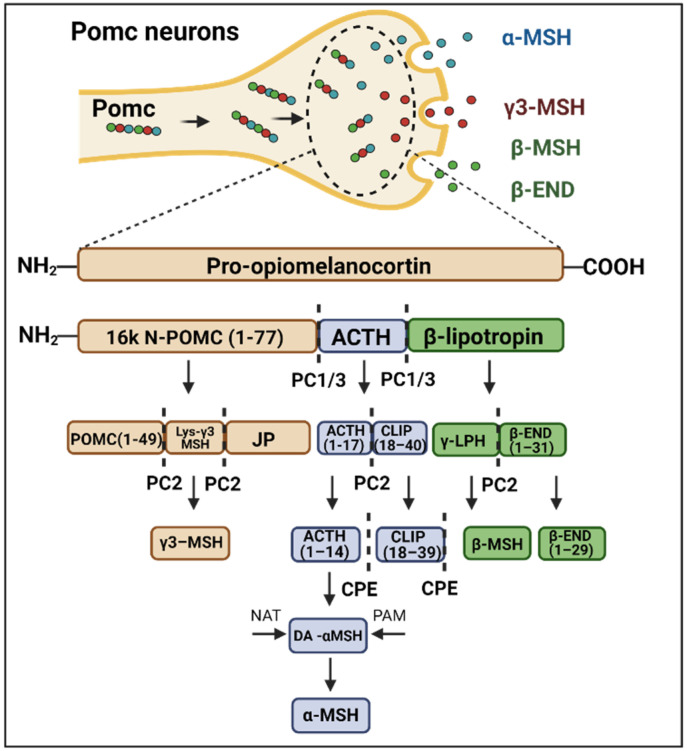
**Schematic representation of Pomc processing**. The balance between food intake and energy expenditure is mediated in part by α-MSH and β-endorphin (β-END) production. Pomc cleavage enzymes such as PC1/3 (convertase protein 1), PC2 (convertase protein 2) and CPE (carboxypeptidase E) act in specific portions of Pomc neuropeptide to catalyze its processing. As a result of this process, there is the production of α-MSH, β-endorphin, β-MSH and γ3-MSH, which are biologically active molecules.

Pomc is a neurotransmitter precursor that undergoes a series of enzyme-catalyzed cleavages, resulting in the production of active neuropeptides [6,7]. A schematic view of the Pomc cleavage process is depicted in Figure 1. Upon the actions of prohormone convertase 1/3 (PC1/3), prohormone convertase 2 (PC2) and carboxypeptidase (CPE), POMC is converted into the active peptides β-lipotropin, adrenocorticotropic hormone (ACTH), α-melanocyte-stimulating hormone (α-MSH), β-melanocyte-stimulating hormone (β-MSH), γ3-melanocyte-stimulating hormone (γ3-MSH), and β-endorphin. In the hypothalamic arcuate nucleus (ARC), α-MSH and β-END are the main products of Pomc involved in energy homeostasis [8,9]. Studies have shown that the consumption of a high-fat diet (HFD), as well as the intracerebroventricular (ICV) injection of palmitate can rapidly impact Pomc processing, resulting in the abnormal regulation of the melanocortin system and favoring body-mass gain [10,11]. At least in part, the dysregulation of Pomc in this context results from the abnormal expression and function of the endopeptidases that catalyze its conversion into the active neuropeptides [10].

The cAMP response element-binding protein (CREB) is one of the transcription factors that control the expression of Pomc endopeptidases [12,13]. CREB phosphorylation in serine-133 induces its translocation to the nucleus and subsequent binding to CRE sites in the promoters of its target genes [12,13]. In the hypothalamus, in response to leptin, cAMP-regulated transcriptional co-activator 1 (CRTC1) promotes satiety as a consequence of CREB-dependent expression of the anorexigenic peptide cocaine-and-amphetamine-related transcript (CART) [14,15]. In addition, single-nucleus RNA sequencing of ARC cells revealed that, in obesity, there is a reduced expression of CREB [16]. However, it is currently unknown if dietary fats have an impact on hypothalamic CREB, leading to an abnormal regulation of the Pomc-processing system. Here, using animal models, we show that hypothalamic CREB is regulated by dietary fats impacting on the expression of a Pomc catalytic enzyme, and ultimately affecting the production of α-MSH.

## 2. Materials and Methods

### 2.1. Experimental Models 

Eight-week-old male C57BL/6 mice were obtained from the animal facility at the University of Campinas and employed in most of the experiments. In some experiments aimed at determining the expression of phospho-CREB (pCREB) and the effects of palmitate on Pomc neurons, Pomc-Cre mice (Tg^(Pomc1-cre)16Lowl^/J, The Jackson Laboratory) were bred with Cre-inducible tdTomato-reporter mice (B6.Cg-Gt(ROSA)26Sor^tm14(CAG-tdTomato)Hze^/J, The Jackson Laboratory) to promote the expression of tdTomato specifically in Pomc neurons (Pomc^tdTomato^ reporter mice); these mutations were confirmed by genotyping the DNA from the tail tip (RED Extract-N-Amp™ Tissue PCR Kit, Sigma-Aldrich, Darmstadt, Germany).

### 2.2. Housing and Diets

In all experiments, mice were maintained under controlled room temperature (22 ± 1 °C) and a 12/12 h dark–light cycle. Standard diet (SD) and water were available ad libitum, except in experimental protocols including fasting and refeeding, which are described as follows: Mice were submitted to an overnight fast, and at 7 am, they were refed with the respective diet for 2 h. Next, mice were fasted again for an additional 4 h; this protocol was employed to synchronize feeding status as previously described [17]. The feeding synchronization paradigms reduce intra- and inter-experimental variability when analyzing parameters related to caloric intake, body mass and metabolism [18,19]. The rationale relies on the fact that mice predominantly feed during the dark cycle and classical overnight fasting protocols disturb the physiological patterns of metabolic regulation; the synchronization protocol mitigates this problem. In some experiments, mice were fed an HFD (composition in Supplementary Table S1). For chronic experiments, mice were randomly divided into five groups: four groups were fed an HFD for 3 days (3D HFD) or 1, 2 or 4 weeks (1 W HFD, 2 W HFD and 4 W HFD, respectively). Each group was paired with a control group (SD) that was exclusively fed an SD throughout the experimental period. A similar protocol was used for acute HFD consumption; however, for this experimental design, mice were fed an HFD for 1, 3, 6 or 9 h (1 H HFD, 3 H HFD, 6 H HFD and 9 H HFD, respectively). Similarly, each group was paired with a control group (SD) that was exclusively fed an SD throughout the experimental period. All experiments were conducted according to the “Guide for the Care and Use of Laboratory Animals of the Institute of Laboratory Animal Resources, US National Academy of Sciences” and approved by the Institutional Animal Care and Use Committee (CEUA 5349-1/2019 and 5575-1/2020) and National Technical Biosafety Committee (CTNBio 7.428/2021) from the University of Campinas, Brazil.

### 2.3. Reagents and Key Resources 

All reagents and key resources used in this study are detailed in Supplementary Table S2.

### 2.4. Stereotaxic Surgery

In order to determine the central effects of sodium palmitate (SP), the mice that were fed an SD were submitted to a fasting and refeeding protocol to synchronize the postprandial status. Both C57BL/6 and Pomc^tdTomato^ mice were anesthetized with ketamine (100 mg/kg) and xylazine (10 mg/kg). To calculate the dosage of anesthesia, we used a smartphone app, *Labsinsane* [20]. All mice were submitted to stereotaxic surgery (Ultra Precise–model 963, Kopf). A total of 2 µL of SP (30 µM) or saline (0.9% NaCl) were injected as a single dose into the lateral ventricle (coordinates from bregma: anteroposterior, −0.94 mm; lateral, −1.5 mm; and depth, −2.2 mm) [21]. After 2 or 4 h, mice were euthanized for tissue harvest. To promote CREB knockdown in the mediobasal hypothalamic area, we tested three commercially available shRNA-lentiviral clones against CREB (Sigma-Aldrich, St Louis, MO, USA; TRCN 96629; TRCN96633 and TRCN 304358); in addition, we employed a scramble sequence experimental control (SHC002V, pLKO.1-puro non-mammalian shRNA control). As TRCN 304358 was proven to be the most efficient, it was employed in all subsequent experiments. Lentiviral shRNA particles (10^6^ TU) were bilaterally injected into the ARC (coordinates from bregma: anteroposterior, −1.7 mm; lateral, ±0.3 mm; and depth, −5.8 mm) of mice; after a period of recovery (10 days), mice were fed an HFD for 3 days (Scramble HFD and shRNA CREB HFD). The control groups were fed an SD throughout the experimental period (Scramble SD and shRNA CREB SD). Body-mass gain and food intake were measured daily for 7 days (day 7 to day 14). In another cohort, mice were submitted to the injection of SP or saline on day 14 (Scramble saline, Scramble SP, shRNA saline and shRNA SP groups), and after 2 h, mice were euthanized for tissue harvest.

### 2.5. Arcuate Nucleus Microdissection

For isolation of the ARC, brains were rapidly harvested and cooled in ice-cold 0.1 M phosphate-buffered saline (PBS) for 5 min, then placed with the ventral surface facing up into a chilled stainless-steel brain matrix (BS-SS 505C, Braintree Scientific, Inc., West Braintree, MA, USA). Next, they were sectioned into coronal slices of 1 mm thickness, and the ARC region was microdissected using a knife cut and a magnifying glass [22]. For analyses, we pooled three hypothalami per sample and analyzed a total of five samples per experimental group (*n* = 5).

### 2.6. Indirect Calorimetry and Measurement of Locomotor Activity

The oxygen consumption (VO_2_), carbon dioxide production (VCO_2_), respiratory exchange ratio (RER), energy expenditure (EE), and total locomotor activity were measured using indirect open-circuit calorimeter system chambers (LE405 Gas Analyzer, Panlab-Harvard Apparatus, Holliston and LE 001 PH Multitake Cage, Panlab-Harvard Apparatus, Holliston, respectively), as previously described [23]. The feeding efficiency was calculated as a ratio between the total body-mass gain (in g) and the cumulative energy intake (in kcal) consumed in the week following the stereotaxic surgery (day 7 to day 14).

### 2.7. Western Blotting

For the protein quantification, whole hypothalamus and dissected ARC samples were homogenized in radioimmunoprecipitation assay buffer (RIPA: 150 mM NaCl, 50 mM Tris, 5 mM EDTA, 1% Triton X-100, 0.5% sodium deoxycholate, and 0.1% sodium dodecyl sulfate that was supplemented with protease inhibitors). Insoluble material was removed by centrifugation (11,000 rpm) for 40 min at 4 °C, and the protein quantification was determined using the supernatant in a biuret protein assay. A total of 100 µg of protein was incubated with Laemmli buffer (0.5 M Tris, 30% glycerol, 10% SDS, 0.6 M DTT, and 0.012 bromophenol blue) for 5 min at 95 °C. The proteins were separated by SDS-PAGE and transferred to nitrocellulose membranes by a Trans-Blot SD Semi-Dry Transfer Cell (Bio-Rad, Hercules, CA, USA) for 1 h at 15 V (constant). Blots were blocked in 5% skimmed milk powder solution in TBST (1 × TBS and 0.1% Tween 20) for 2 h at RT, and incubated with the primary antibodies pCREB (1:1000; CS #9198) and α-tubulin (1:5000; ab7291) overnight at 4 °C. HRP-coupled secondary antibodies (1:5000) were used for detection of the chemiluminescence, and visualization was achieved by exposure to an Image Quant LAS4000 camera (GE Healthcare, Life Sciences, Piscataway, NJ, USA). The bands were quantified by densitometry using Image J software (National Institutes of Health). Raw data regarding blots are depicted in Supplementary Tables S3–S8.

### 2.8. RNA Extraction and Quantitative Real-Time PCR

Total RNA was extracted from whole hypothalamus and dissected ARC samples using TRIzol reagent (Invitrogen, Waltham, MA, USA), according to the manufacturer’s recommendations. A total of 2 µg of RNA were employed for the synthesis of complementary DNA (cDNA), using the High-Capacity cDNA Reverse Transcription Synthesis Kit (Applied Biosystems). Gene expression analysis was performed via RT-qPCR using TaqMan Universal PCR Master Mix (7500 detection system, Applied Biosystems), the following primers were used: *Pomc* (Mm00435874_m1), *Cart* (Mm01811203_g1), *AgRp* (Mm00475829_g1), *Npy* (Mm01410146_m1), *Pcsk1* (Mm01345253_m1), *Pcsk2* (Mm00500981_m1), *Prcp* (Mm00804502_m1), *Cpe* (Mm00516341_m1), *Il1β* (Mm00434228_m1), *Il6* (Mm00446190_m1), *Il10* (Mm01288386_m1), *Tgfβ1* (IDT 138333258), *Tlr4* (Mm00445273_m1), *Tnf* (Mm00443258_m1), *Nlrp3* (Mm00840904_m1) and *Socs3* (Mm00545913_s1). *Gapdh* (Mm99999915_g1) was employed as a reference gene. 

### 2.9. Heart Perfusion and Immunofluorescence Assay 

Mice were deeply anesthetized with xylazine and ketamine, as previously described, and the left cardiac ventricle was thoroughly perfused with saline (0.9% NaCl), followed by 4% paraformaldehyde (PFA). The whole brains were removed and post-fixed for 24 h in PFA and dehydrated in a buffer containing 30% sucrose at 4 °C. Serial brain coronal slices (30 µm) were obtained in a cryostat (LEICA Microsystems, CM1860) and stored in anti-freezing solution. For the free-floating immunostaining, slices were blocked with 0.25% Triton X-100 and 5% donkey serum in 0.1 M PBS for 2 h at RT, followed by overnight incubation at 4 °C with the primary antibody pCREB (1:500; CS #9198) in the blocking solution. Next, the sections were incubated with the secondary antibody donkey anti-rabbit FITC (1:500; ab6798) in the blocking solution for 2 h at RT, and then mounted using an antifade mountain medium with DAPI (4′,6-diamidino-2-phenylindole) Vectashield^®^ (Vector Laboratories H-1200-10). For identification of Pomc neurons that co-expressed pCREB, we analyzed immunostained images from the hypothalamus region (1.06 to 2.30 mm posterior to Bregma) of Pomc^tdTomato^ mice in basal conditions. The number of Pomc neurons, pCREB-expressing cells and colocalization were determined and expressed as percentages. All the samples were examined at the National Institute of Science and Technology on Photonics Applied to Cell Biology (INFABIC) of the State University of Campinas, using a Zeiss LSM 780-NLO confocal microscope (Zeiss, Oberkochen, Germany).

### 2.10. Stereological Analysis

A stereology method was employed to analyze the expression of pCREB in Pomc neurons after saline or SP stimulus in Pomc^tdTomato^ mice. Systematic sampling of every fourth section was collected through the ARC (from rostral to caudal axis), starting at −1.22 mm (bregma) and ending at −2.54 mm. Five slices were used for the stereological analysis of each mouse. Double-labeling was determined using a z-series of confocal microscopy covering all Pomc neurons within the ARC. Next, all images were analyzed using an automated method by Image J plugging, Coloc-2. The ARC region was exclusively selected using a region of interest (ROI), and the number of double-labeling neurons was quantified in all slices of the z-stack acquisition. The number of double-labeled cells was expressed by Pearson’s correlation and validated by the Costes correlation, as previously described [24]. The images were acquired using a confocal microscope (Zeiss Upright LSM780 NLO) from the National Institute of Science and Technology on Photonics Applied to Cell Biology (INFABIC) at the University of Campinas.

### 2.11. Determination of Neuropeptides

Serum concentrations of α-MSH and β-endorphin were assessed by an immunoassay (Milliplex^®^ #RMPPMAG-83K), according to the manufacturer’s recommendations. The absorbances of the samples were obtained by the Bio-Plex 200 system (Bio-Rad) at the Life Sciences Core Facility (LaCTAD) at the University of Campinas.

### 2.12. Analysis of Single-Cell RNA Sequencing Data

Public single-cell RNA sequencing (scRNAseq) data from the mouse arcuate nucleus and median eminence (Arc-ME) comprising 20,921 cells [22] were retrieved for this study. For the visualization of these data, the unsupervised machine-learning algorithm called diffusion-based Manifold Approximation and projection (dbMAP) [25] was used. Briefly, dbMAP encodes expression data in an adaptive diffusion map prior to graph layout optimization with an adapted UMAP [26] algorithm. Gene expression was then visualized using Seurat v3 [27] with default parameters. To detect *Creb1* transcriptional targets in the Arc-ME, this data was analyzed with the pySCENIC workflow program [28]. Briefly, pySCENIC applies arboreto [29] to estimate the gene regulatory networks (GRNs) contained in single-cell expression data, building a reference of regulons (transcriptional factors and their associated targets). This reference is then scored against databases of known motifs, enhancers and promoter regions so as to filter eventual spurious results. The *Creb1* regulon was then subset and its transcriptional targets were ranked by their relative importance (as per arboreto adjacency matrix). A value of 1.0 relative importance was used as the significance threshold, resulting in the identification of 186 significative targets. These are included in Supplementary Table S9 and visualized by their relative importance.

### 2.13. Statistical Analysis 

Data are expressed as the means ± standard deviation (SD) or standard errors of the means (SEMs) whenever appropriate and as indicated in the results section and figure legends. The number of independent experiments is indicated in the figures. For statistical analyses, groups were compared using unpaired two-tailed Student’s *t*-test, and one-way or two-away analysis of variance (ANOVA) when appropriate. Post hoc comparisons were performed using Tukey’s or Sidak’s test to determine the significance difference. For the stereological analysis, Pearson and Costes correlations were used. A *p*-value ≤ 0.05 was considered statistically significant. GraphPad Prism 8.0 was used for the statistical analysis.

## 3. Results

### 3.1. Identifying the Transcriptional Targets for CREB in the Hypothalamic ARC

**Figure 2:**
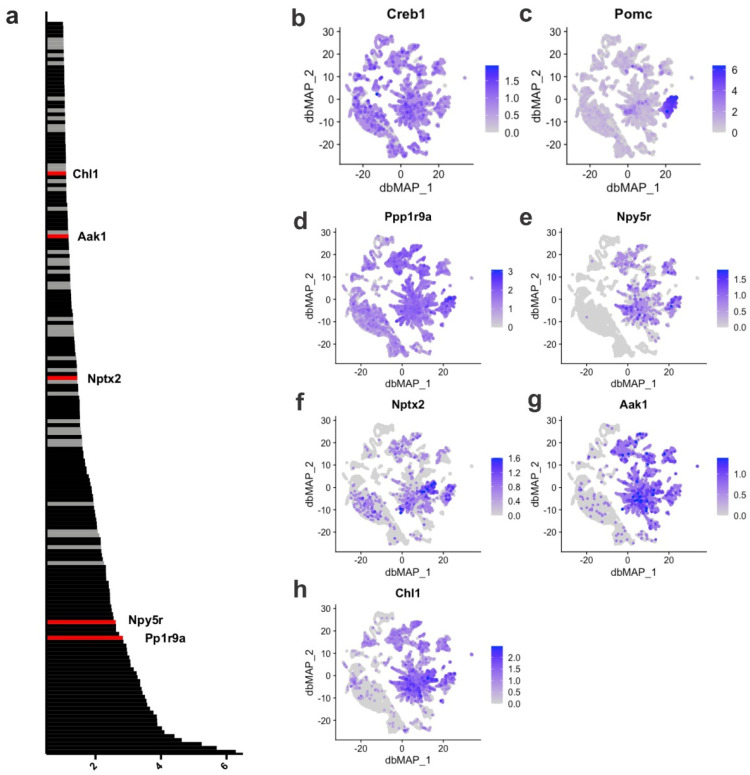
**Creb1 transcriptional targets in the Arc-ME**. (**a**) Relative importance scores for each of the 186 significant Creb1 transcriptional targets; (**b**) gene expression of *Creb1* across cells in the Arc-ME; (**c**) gene expression of Pomc across cells in the Arc-ME. Based on their expression in Pomc-expressing cells, those with higher expression are highlighted in red and have their gene expression displayed beside: (**d**) *Ppp1r9a*; (**e**) *Npy5r*; (**f**) *Nptx2*; (**g**) *Aak1* and (**h**) *Chl1*. In contrast, those with lower/no expression in these cells have their scores represented in gray. Gene symbols, relative importance scores and functions are available in Supplementary Table S2.

To identify the transcriptional targets for CREB, we estimated the GRNs of the neuronal subpopulations of the ARC and their associated regulons (transcriptional factors and targets). Initially, we identified 186 potential target genes for CREB (Supplementary Table S9). We found that five of these genes are targets for CREB activation that are expressed at high levels in Pomc neurons (Figure 2), including Ppp1r9a, Npy5r, Nptx2, Ask1, and Chl1, which are abundantly expressed in the ARC (Figure 2); however, they are not the ones with the greatest expression in the ARC (Supplementary Figure S1). These downstream validated targets were also identified, presenting either direct or indirect correlation with CREB in the hypothalamus and specifically in Pomc neurons. The functions of several important targets of CREB identified by both approaches are listed in Supplementary Table S3 and include genes involved in neuronal growth, plasticity and differentiation, metabolism, DNA repair, and neuroendocrine regulation.

### 3.2. Hypothalamic CREB Is Expressed in Pomc Neurons and Is Modulated in Response to a High-Fat Diet

**Figure 3:**
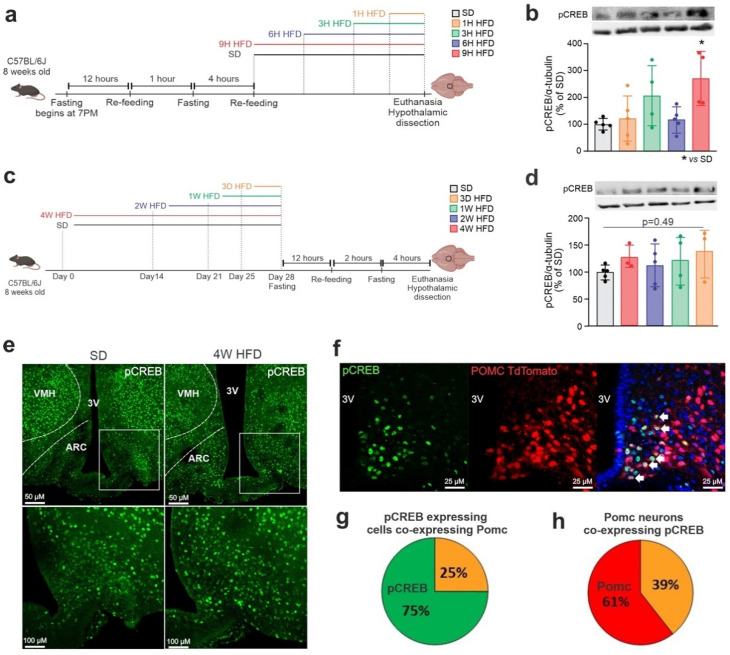
**HFD modulates pCREB expression and distribution in the hypothalamus.** Adult male C57BL/6J mice were fed on an HFD for chronic and acute periods. (**a**) Schematic representation of the experimental protocol of HFD-acute feeding; (**b**) Western blotting of the hypothalamus after an acute period of HFD. (**c**) Schematic representation of the experimental protocol of HFD-chronic period; (**d**) Western blotting of the hypothalamus after a chronic period of HFD; (**e**) coronal brain sections of mediobasal hypothalamus of mice fed an SD and 4 weeks of HFD were immunostained for pCREB; the anatomical boundaries are depicted. 3V, third ventricle; ARC, arcuate nucleus; VMH, ventromedial hypothalamus; (**f**) coronal brain sections of Pomc^tdTomato^ were immunostained for pCREB. Quantification of double-labeled cells; (**g**) number of pCREB-expressing cells co-expressing Pomc; and (**h**) number of Pomc neurons co-expressing pCREB. In (**g**,**h**), orange represents colocalization. Data are presented as means ± SD. *n* = 4–5 per group. One-way ANOVA followed by Tukey’s post hoc test used for statistical analyses. * *p* ≤ 0.05 in comparison with the SD group.

To determine whether CREB is modulated in the hypothalamus in response to the consumption of an HFD, mice were submitted to the protocols depicted in Figure 3a,c, and hypothalami were harvested for immunoblotting experiments. As shown in Figure 3b,d, there was an increased phosphorylation of hypothalamic CREB 9 h after the introduction of an HFD. Next, immunofluorescence experiments revealed that the consumption of an HFD affected the anatomical distribution of pCREB in the hypothalamus; thus, in mice fed an HFD, there was a reduction in pCREB in the ventromedial hypothalamus (VMH) and an increase in the ARC (Figure 3e). In addition, there was an overlap of pCREB with Pomc neurons in the ARC of Pomc^tdTomato^ mice, where of all the pCREB expression, 25% is in Pomc neurons (Figure 3g) and 39% of Pomc neurons express pCREB (Figure 3h), indicating that diet-induced changes in ARC pCREB distribution could impact Pomc neuronal activity (Figure 3f).

### 3.3. ARC CREB Regulation in Response to Dietary Intervention

**Figure 4:**
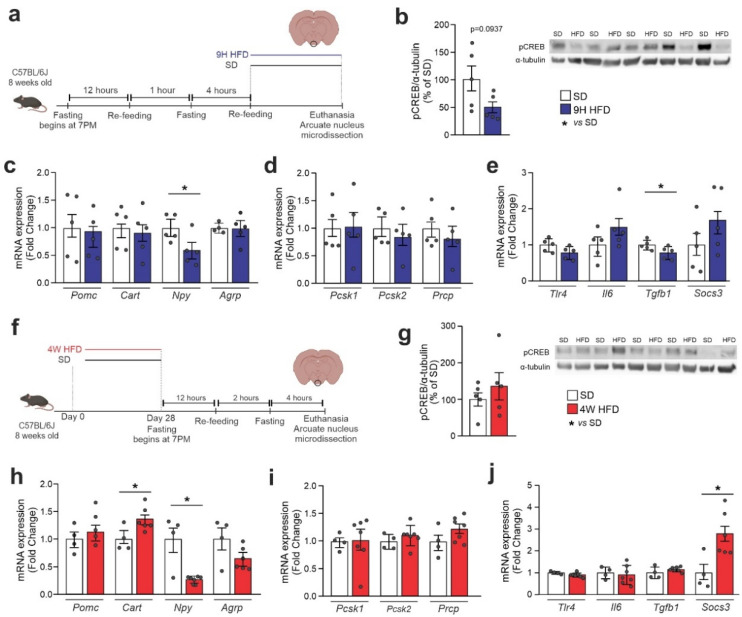
**CREB^ARC^ expression is modulated upon HFD.** Adult male C57BL/6J mice were fed an HFD for chronic and acute periods and the arcuate nucleus (ARC) was microdissected. (**a**) Schematic representation of the experimental protocol of HFD-acute feeding; (**b**) Western blotting of the ARC after an acute period of HFD; ARC mRNA levels of (**c**) neuropeptides; (**d**) Pomc-related processing enzymes and (**e**) inflammatory markers. (**f**) Schematic representation of the experimental protocol of HFD-chronic feeding; (**g**) Western blotting of the ARC after a chronic period of HFD; ARC mRNA levels of (**h**) neuropeptides, (**i**) Pomc-related processing enzymes and (**j**) inflammatory markers. Data are presented as means ± SEM. We pooled ARC from three mice per sample. *n* = 5 per group. Student’s *t*-test was used for statistical analyses. * *p* ≤ 0.05 in comparison with the SD group.

Because, in the immunofluorescence experiments, we observed that the consumption of an HFD resulted in the change in hypothalamic pCREB anatomical distribution, we decided to specifically dissect the ARC to determine the impact of distinct dietary interventions in the activation of CREB. For that, mice were initially submitted to a short-term HFD protocol, as depicted in Figure 4a, and at the end of the experimental intervention, the ARC was microdissected for further analysis. As shown in Figure 4b, there was a trend to reduce pCREB in mice that were fed an HFD. This was accompanied by a reduction in Npy (Figure 4c), no modification in the expression of Pomc endopeptidases (Figure 4d), and a reduction in Tgfb1 (Figure 4e). Next, we evaluated microdissected ARCs from mice fed an HFD for 4 weeks (Figure 4f). Under this experimental condition, there was a trend toward increasing pCREB in the ARC (Figure 4g), recapitulating the results obtained in the immunofluorescence experiments (Figure 3e). This was accompanied by a reduction in the expressions of CART and Npy (Figure 4h), no changes in Pomc endopeptidases (Figure 4i), and increased Socs3 (Figure 4j).

### 3.4. Hypothalamic CREB Is Activated by Palmitate

**Figure 5:**
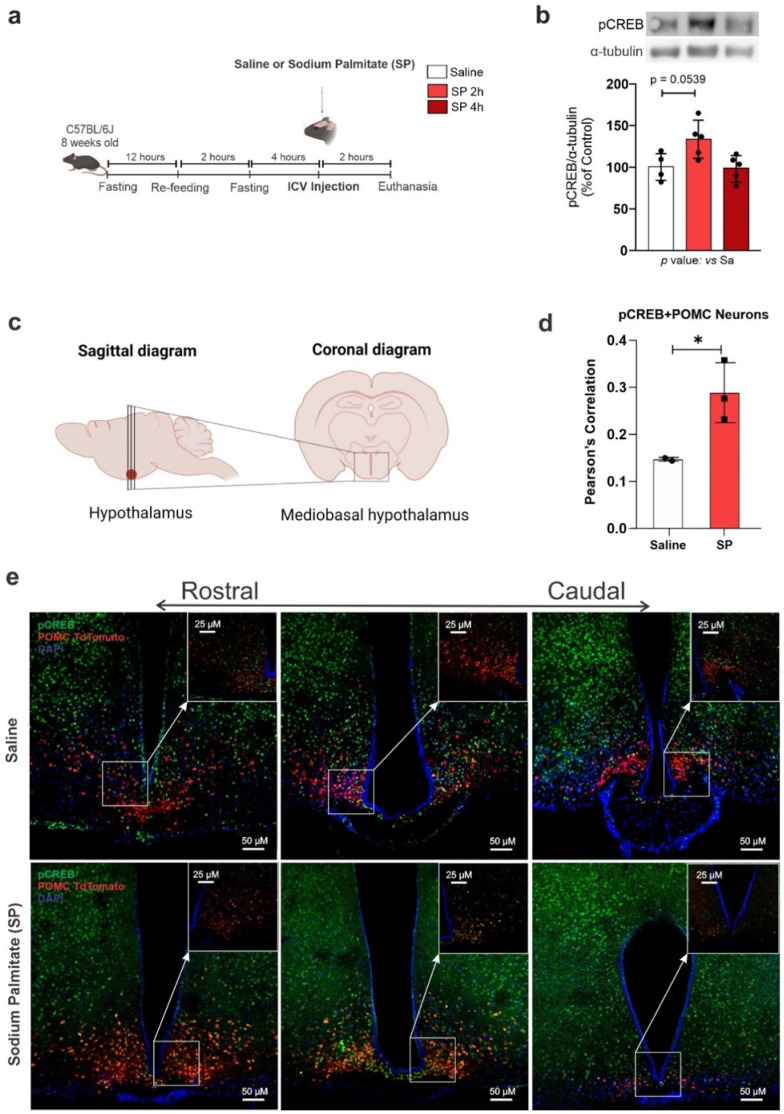
**Palmitic acid modulates hypothalamic CREB expression specifically in Pomc neurons.** Adult male C57BL/6 and Pomc^tdTomato^ mice were submitted to an ICV injection of saline or 30 μM sodium palmitate (SP) and the hypothalamus was collected at different time points after injection. (**a**) Schematic representation of the experimental protocol; (**b**) Western blotting of the hypothalamus; (**c**) schematic representation of stereological protocol; (**d**) quantification of double-labeling neurons; (**e**) coronal brain sections of the mediobasal hypothalamus were immunostained with pCREB; the arrows indicate higher magnification of the selected region. For Western blotting, data are presented as means ± SD. *n* = 4–5 per group. ANOVA followed by Tukey’s post hoc test was used for statistical analyses. * *p* ≤ 0.05 in comparison with the saline group. For immunostaining data are presented as means ± SD. *n* = 2–3. Pearson’s correlation followed by Costes correlation was used for statistical analyses. * *p* ≤ 0.05 in comparison with the saline group.

In human Pomc neurons, palmitate is known to modulate Pomc-processing enzymes and the production of α-MSH and β-endorphin [10]. Here, in mice submitted to acute ICV injections of palmitate (Figure 5a), there was an increase in hypothalamic pCREB 2 h after injection (Figure 5b). Next, we employed stereological analysis to determine the impact of an acute ICV injection of palmitate on the activation of CREB, specifically in Pomc neurons. For that, Pomc^tdTomato^ mice that were exclusively fed an SD were treated with a single dose of palmitate injected directly into the lateral ventricle, and after 2 h, the brain was extracted and sectioned, as shown in Figure 5c. A stereological analysis demonstrated a 1.96-fold increase in pCREB in Pomc neurons (Figure 5d,e).

### 3.5. The Knockdown of Hypothalamic CREB Worsens the HFD-Induced Metabolic Phenotype

**Figure 6:**
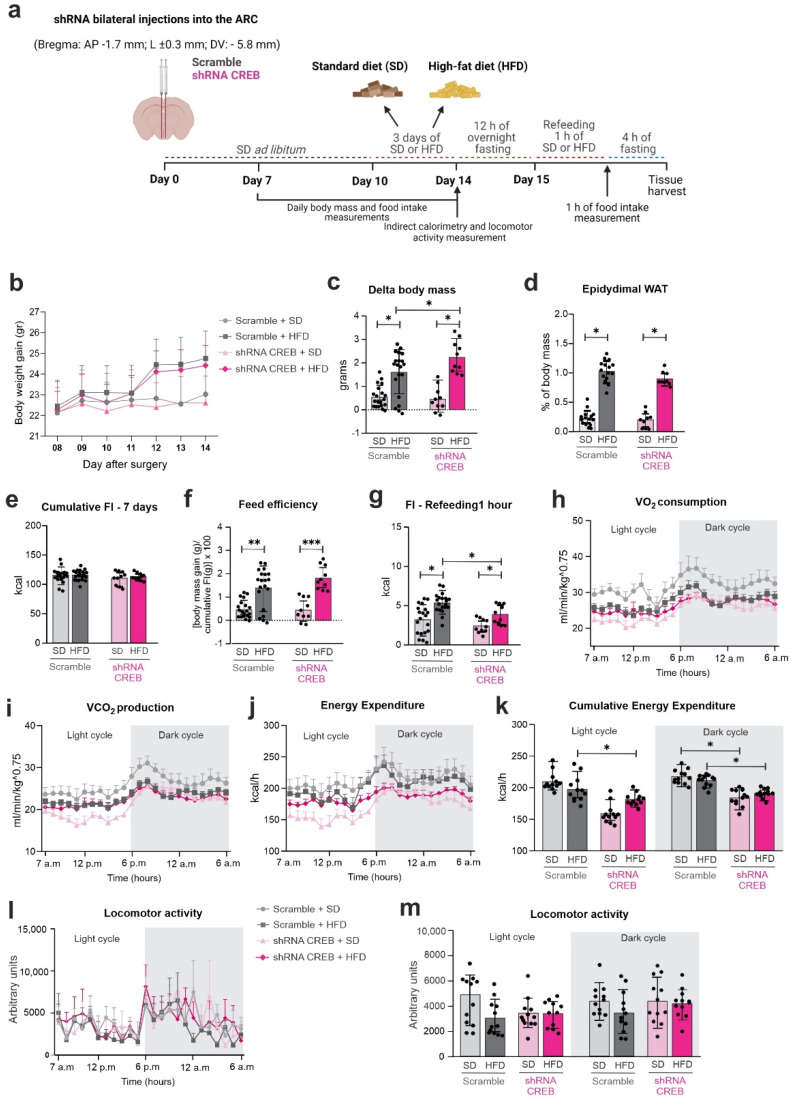
**Hypothalamic CREB down-regulation changes metabolic parameters upon short-term HFD**. Adult male C57BL/6J mice were submitted to a bilateral injection of lentivirus particles (Scramble or shRNA CREB) into the arcuate nucleus (ARC) and, after 10 days, were fed an SD or HFD for 3 days. Hypothalamus and blood were collected at day 15 after surgery. (**a**) Schematic representation of experimental protocol; (**b**) body-mass gain; (**c**) delta body mass; (**d**) epididymal fat depot; (**e**) cumulative food intake; (**f**) food intake over an hour after a 12 h fasting; (**g**) feed efficiency; (**h**) oxygen (O_2_) consumption; (**i**) carbon dioxide (CO_2_) production; (**j**) energy expenditure; (**k**) cumulative energy expenditure; and (**l**,**m**) total locomotor activity in metabolic cage during light and dark cycles on day 10 post-surgery. Data are presented as means ± SD. *n* = 15–20 mice in two different cohorts. Two-way ANOVA followed by Sidak’s post hoc test was used for statistical analyses. * *p* ≤ 0.05, ** *p* < 0.01 and *** *p* < 0.001 in comparison with groups indicated in the figure.

To determine the impact of hypothalamic CREB inhibition, we performed bilateral injections of lentiviral shRNA particles into the ARC that were designed to inhibit the expression of CREB. The experimental protocol is depicted in Figure 6a. We tested three distinct lentiviral sequences for CREB inhibition (Supplementary Figure S2a); the most efficient sequence (TRCN304358) reduced CREB expression in the hypothalamus by 50%, which was confirmed by immunofluorescence assay (Supplementary Figure S2b) and PCR (Supplementary Figure S2c); this lentiviral sequence was used in all the subsequent knockdown experiments. The knockdown of hypothalamic CREB resulted in increased body-mass gain (Figure 6b,c), without affecting epidydimal white adipose tissue depots (Figure 6d), cumulative food intake (Figure 6e) or feed efficiency (Figure 6f). However, there were significant reductions in short-term refeeding (Figure 6g) and in 24 h energy expenditure (Figure 6h–k), without affecting locomotor activity (Figure 6l,m). 

### 3.6. Hypothalamic CREB Knockdown Reduces Pomc-Related Processing Enzymes and Affects α-MSH Levels

**Figure 7:**
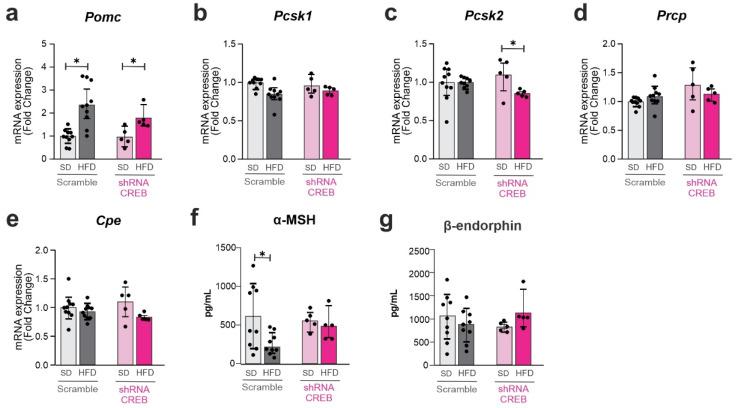
**The impact of hypothalamic CREB on *Pomc* cleavage enzymes upon short-term HFD.** Adult male C57BL/6J mice were submitted to a bilateral injection of lentivirus particles (Scramble or shRNA CREB) into the arcuate nucleus (ARC) and, after 10 days, were fed an SD or HFD for 3 days. Hypothalamus and blood were collected at day 15 after surgery. (**a**–**e**) Hypothalamic mRNA expression of Pomc and Pomc-related processing enzymes; (**f**,**g**) serum levels of Pomc peptide products. Data are presented as means ± SD. *n* = 5–15 mice in two different cohorts. Two-way ANOVA followed by Sidak’s post hoc test was used for statistical analyses. * *p* ≤ 0.05 in comparison with groups indicated in the figure.

The inhibition of hypothalamic CREB promoted no change in Pomc (Figure 7a), Pcsk1 (Figure 7b), Prcp (Figure 7d) or CPE (Figure 7e); however, there was a reduction in Pcsk2 (Figure 7c). This was accompanied by a loss of the effect of an HFD reducing hypothalamic α-MSH (Figure 7f) and by no modification of β-endorphin (Figure 7g). Moreover, the inhibition of hypothalamic CREB promoted an increase in hypothalamic Tgfb1 (Supplementary Figure S3g), an increase in baseline Agrp (Supplementary Figure S3h), and an increase in CART (Supplementary Figure S3i).

### 3.7. Hypothalamic CREB Knockdown Reverses the Effects Promoted by Palmitate

**Figure 8:**
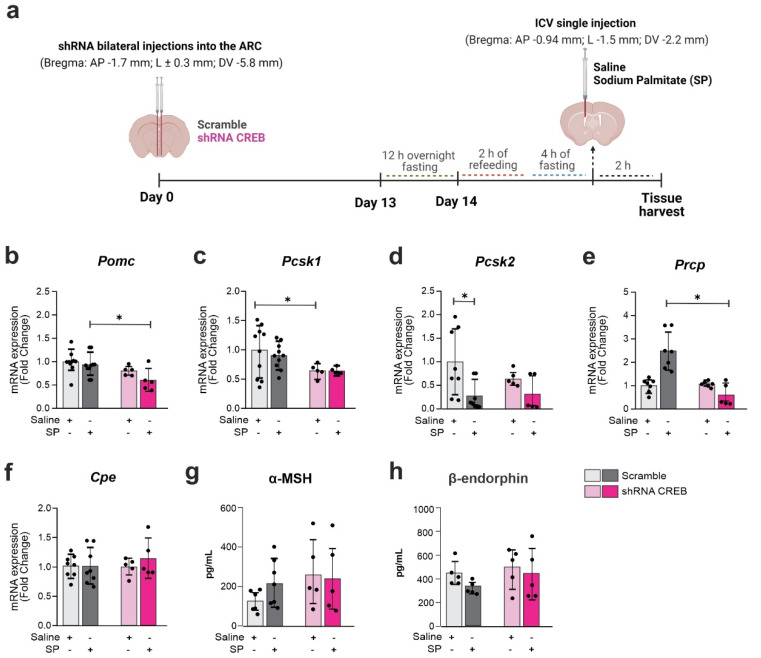
**Reduced CREB expression changes *Pomc* cleavage enzymes expression upon palmitic acid stimulus.** Adult male C57BL/6J mice were submitted to a bilateral injection of lentivirus particles (Scramble or shRNA CREB) into the arcuate nucleus (ARC) and, after 14 days, were ICV injected with saline or sodium palmitate (SP) 30 µM. (**a**) Schematic representation of experimental protocol; (**b**–**f**) Hypothalamic mRNA expression of Pomc and Pomc-related processing enzymes; (**g**,**h**) serum levels of Pomc peptide products. Data are presented as means ± SD. *n* = 5–8 mice. Two-way ANOVA followed by Sidak’s post hoc test was used for statistical analyses. * *p* ≤ 0.05 in comparison with groups indicated in the figure.

Next, we evaluated the impact of hypothalamic CREB knockdown on the regulation of the effects of palmitate in the hypothalamus. For that, C57Bl/J6 mice were submitted to SP infusion in the third ventricle according to the experimental design depicted in Figure 8a. Two hours later, the hypothalamus was dissected and the Pomc cleavage enzymes were analyzed. The inhibition of CREB resulted in no changes in the hypothalamic expressions of CPE (Figure 8f), α-MSH (Figure 8g) or β-endorphin (Figure 8h). However, there were reductions in the palmitate-stimulated levels of Pomc (Figure 8b) and baseline levels of Pcsk1 (Figure 8c); there was also a loss of the effect of palmitate reducing Pcsk2 (Figure 8d) and a reduction in Prcp in the palmitate-treated groups (Figure 8e). Regarding the inflammatory markers, under the hypothalamic inhibition of CREB, palmitate induced a reduction in baseline IL-6 (Supplementary Figure S4d), and an increase in Socs3 (Supplementary Figure S4f). 

## 4. Discussion

This experimental study has shown that the consumption of an HFD promotes the activation of CREB in ARC Pomc neurons and that, upon CREB inhibition, there is increased diet-induced body-mass gain, a reduction in energy expenditure and a reduction in the expression of Pomc endopeptidases, affecting the production of the Pomc subproduct, α-MSH.

The hypothalamic melanocortin system plays a central role in the control of caloric intake and energy expenditure [30,31]. ARC Pomc neurons are responsive to hormones, such as leptin, insulin and GLP1, and to nutrients, such as glucose, amino acids and fatty acids, which act as signals that indicate the energy status of the organism [32]. Once active, Pomc neurons connect to second-order neurons to control food intake, energy expenditure and systemic metabolism [32,33]. There are two subproducts of Pomc that act as neurotransmitters to deliver energy homeostatic signals: α-MSH and β-endorphin [3,9,32]. The productions of α-MSH and β-endorphin are finely tuned by a series of endopeptidases that catalyze the cleavage of Pomc [32] (Figure 1). Studies have shown that dietary fats can affect the expression of Pomc endopeptidases, thereby impacting the production of its subproducts [10,34]. 

CREB is one of the transcription factors that control the expression of Pomc endopeptidases [35]. In a single-cell transcriptomics analysis of the hypothalamus, it has been shown that, in long-term diet-induced obesity, the expression of CREB is reduced in AgRP/NPY neurons [16]; however, no previous study has evaluated the impact of the short-term consumption of an HFD on Pomc CREB. In addition, the main transcriptional targets of CREB, specifically in ARC Pomc neurons, have not been elucidated. Combining the analysis of ARC single-cell transcriptomics and gene regulatory networks, we identified five transcriptional targets of CREB that are predominantly expressed in Pomc: *Ppp1r9a*, *Npy5r*, *Nptx2*, *Ask1* and *Chl1. Ppp1r9a* encodes for protein phosphatase 1 regulatory subunit 9A, which is involved in actin filament cross-linking, particularly in neurite formation [36]. Studies have shown that *Ppp1r9a* is an imprinted gene that undergoes regulation under overfeeding conditions, which could impact metabolic control [37]. *Npy5r* encodes for NPY receptor Y5, and there is large amount of data exploring its role in food intake and energy metabolism [38,39]. *Nptx2* encodes for neuronal pentaxin 2, which is involved in excitatory synapse formation [40]. Studies have provided evidence for the association of Nptx2 abnormalities and sleep disorders, which are highly associated with obesity [41,42]. *Ask1* encodes for mitogen-activated protein kinase 5, a ubiquitous protein that belongs to the MAP kinase signal-transduction system, a well-known component of the system that regulates hypothalamic neurons involved in the control of feeding [43,44]. *Chl1* encodes for cell adhesion molecule L1-like, which is involved in neural cell adhesion [45]. There are no data reporting the association of *Chl1* with hypothalamic function or obesity. These data provide a new window of opportunity to explore the mechanisms involved in CREB regulation of Pomc neurons and their potential involvement in obesity and metabolic disorders.

In addition to the identification of previously unknown hypothalamic CREB transcriptional targets, we provided an advancement of the understanding of how food intake and nutrients regulate hypothalamic CREB. First, using immunoblot, we showed that the hypothalamic expression of pCREB undergoes a rapid and transient increase following the introduction of an HFD. However, when we used immunofluorescence to determine the distribution of pCREB in the hypothalamus, it was clear that the dietary intervention promoted different regulations of pCREB depending on the nuclei. Particularly, in the ARC there was an increase in pCREB even after four weeks on an HFD. Using a stereological analysis, we showed that palmitate promotes the activation of CREB in ARC Pomc neurons and this is accompanied by an increased expression of CART, which is co-expressed in Pomc neurons. Our findings are in line with previous studies that have shown that CREB is involved in the regulation of CART gene expression [46], and that CREB, at least in part, mediates the actions of leptin to induce the expression of CART [47]. 

Palmitate is one of the most important components of an HFD. It is also the predominant fatty acid consumed by humans [48,49,50]. Here, we showed that palmitate injected directly into the hypothalamus of mice promoted the increased activation of CREB, which predominantly occurred in Pomc neurons. This is the first evidence supporting an effect of a highly consumed saturated fat in the regulation of hypothalamic CREB. As previous studies were mostly focused on leptin [51,52] and NPY [53] as modulators of hypothalamic CREB, our finding extends the current knowledge, showing that a nutrient is also capable of regulating hypothalamic CREB. This is in line with data showing that CREB integrates a nutrient-sensing system in the hypothalamus of fish [54]. 

In order to explore the involvement of hypothalamic CREB in energy homeostasis, we site-specifically inhibited CREB expression and determined the impact of this approach on two distinct settings: feeding on an HFD and injection of palmitate into the hypothalamus. In the case of feeding on an HFD, the inhibition of hypothalamic CREB resulted in increased body-mass gain, which was mostly due to reduced energy expenditure. This phenotypic outcome was accompanied by changes in the expressions of the enzymes involved in Pomc processing and in the regulation of one subproduct of Pomc, α-MSH. At least in part, the effects of inhibiting hypothalamic CREB in mice that were fed an HFD were reproduced in the context of palmitic acid injection into the hypothalamus, thus providing further support for the role of CREB as a component of the hypothalamic system involved in the sensing of nutrients [54,55,56]. It is worthwhile mentioning that the enzymatic processing of Pomc depends on multiple enzymes acting in distinct substrates. Thus, even when mapping the detailed changes in the amounts of each of the enzymes involved in this process, it is still impossible to predict the outcome regarding the amounts of the products α-MSH and β-endorphin.

We acknowledge the study has some weaknesses that could be addressed in future work. i, As previously reported [34,57], mice fed on an HFD present considerable variability regarding caloric intake, body-mass variation and other metabolic parameters, which could have impacted the statistical analysis of some of the parameters evaluated in this study. ii, There were differences in the outcomes related to CREB inhibition when comparing the experiments performed with mice fed on an HFD versus experiments using the palmitate paradigm. Palmitate is commonly used in experimental work to explore the role of saturated fats in distinct metabolic conditions; nevertheless, despite the fact that palmitate is the most common fat consumed in the human diet, it is not the only one. So, differences found when comparing the impact of an HFD versus palmitate could be attributed to other nutritional components of the diet. iii, The immunofluorescence experiments revealed that CREB is expressed in distinct subpopulations of cells in the hypothalamus; the different patterns of regulation of CREB under the interventions tested in this study, namely HFD and palmitate, suggest that CREB exerts different functions in each cell type. Thus, future studies should define the functions of CREB in each subpopulation in order to provide a more detailed view of hypothalamic CREB functions. 

## 5. Conclusions

In conclusion, this study has identified CREB as a transducer of signals that control the activity of hypothalamic neurons involved in the regulation of whole-body energy stores. The inhibition of hypothalamic CREB resulted in the abnormal regulation of the Pomc-processing enzyme Pcsk2 and an abnormal regulation of the α-MSH response to an HFD, which was accompanied by a reduction in energy expenditure (graphical abstract). Thus, hypothalamic CREB emerges as a dietary fat-sensitive pathway that could be further explored in studies aimed at advancing the understanding of the pathogenetic mechanisms responsible for the development of diet-induced obesity.

## Data Availability

Source code and materials for reproducing the exposed bioinformatic analyses are available at https://github.com/OCRC/Zanesco_et_al_2022. Raw data for all experiments are available at: Ariane Maria Zanesco, 2022, “Hypothalamic CREB Regulates the Expression of Pomc-Processing Enzyme Pcsk2”, https://doi.org/10.25824/redu/WMNTS8, Repositório de Dados de Pesquisa da Unicamp, DRAFT VERSION, UNF:6:XUe3jTfJLbHwnBulY7VwDQ== [fileUNF].

## Supplementary Materials

The following supporting information can be downloaded at: https://www.mdpi.com/article/10.3390/cells11131996/s1, Figure S1: Major Creb1 transcriptional targets in the Arc-ME. Gene expression of (a) *Ism1*; (b) *Atp6v1c2*; (c) *Ccnd2*; (d) *Ccdc39*; (e) *Stc1*; (f) *Mgat3*; (g) *Gmcl1*; (h) *Pik3r2*; (i) *Utp20*; (j) *Asic1*; (k) *Usp45* and (l) *Hspbap1*, the 12 major Creb1 transcriptional targets with higher relative importance (excluding unclassified/predicted genes). Figure S2: Characterization of hypothalamic CREB knockdown. Adult male C57BL/6J mice were submitted to bilateral injection of lentivirus particles (Scramble and shRNA CREB) into the arcuate nucleus (ARC). Three distinct lentiviruses were tested for their efficiency to inhibit the expression of hypothalamic CREB. (a) Western blot of hypothalamic samples obtained 14 days after CREB down-regulation with different lentiviral-shRNA sequences; (b) Coronal brain section of mediobasal hypothalamus 14 days after the bilateral injection of shRNA CREB (TRCN304358) and Scramble particles into the ARC were immunostained with pCREB; the confocal microscope acquisition settings were kept the same for both groups. Hypothalamic mRNA expression of Creb in different conditions; (c) Adult male C57BL/6J mice were submitted a bilateral injection of lentivirus particles (Scramble or shRNA CREB) into the arcuate nucleus (ARC) and, after 10 days, were fed on SD or HFD for 3 days. Data are presented as means ± SEM. Two-way ANOVA followed by Sidak’s post hoc test was used for statistical analyses. * *p* ≤ 0.05 in comparison with groups indicated in the figure. Figure S3: Hypothalamic CREB down-regulation slightly modulates inflammatory markers and neuropeptides. Adult male C57BL/6J mice were submitted to bilateral injection of lentivirus particles (Scramble and shRNA CREB) into the arcuate nucleus (ARC) and, after 10 days, were fed on SD or HFD for 3 days. (a) Schematic representation of experimental protocol; mRNA gene expression of inflammatory (b–g) and neuropeptides (h–i). Data are presented as means ± SEM. *n* = 5–15 mice in two different cohorts. Two-way ANOVA followed by Sidak’s post hoc test was used for statistical analyses. * *p* ≤ 0.05 in comparison with groups indicated in the figure. Figure S4: Hypothalamic CREB knockdown does not modulate inflammatory markers upon palmitic acid stimulus. Adult male C57BL/6J mice were submitted a bilateral injection of lentivirus particles (Scramble or shRNA CREB) into the arcuate nucleus (ARC) and, after 14 days, were ICV injected with saline or sodium palmitate (SP) 30 µM. (a) Schematic representation of experimental protocol, hypothalami were harvested 2 hours after injection. (b–f) mRNA inflammatory markers. Data are presented as means ± SEM. *n* = 5–15 mice in two different cohorts. Two-way ANOVA followed by Sidak’s post hoc test was used for statistical analyses. * *p* ≤ 0.05 in comparison with groups indicated in the figure. Table S1: Macronutrient composition of the diets. Table S2: Key resources table. Table S3: Quantitative analysis of western blot presented in Figure 3B. Table S4: Quantitative analysis of western blot presented in Figure 3D. Table S5: Quantitative analysis of western blot presented in Figure 4B. Table S6: Quantitative analysis of western blot presented in Figure 4G. Table S7: Quantitative analysis of western blot presented in Figure 5B. Table S8: Quantitative analysis of western blot presented in Supplementary Figure S2a. Table S9, accompanying Excel file: Potential targets for CREB, as determined with the pySCENIC workflow program.
