# Supplementary material for: Hypothalamic CREB Regulates the Expression of Pomc-Processing Enzyme Pcsk2
Source: Cells. 2022 Jun 22;11(13):1996. doi: 10.3390/cells11131996 (PMC9265861; doi:10.3390/cells11131996)
Supplement: Supplementary file 1 [file cells-11-01996-s001.zip › cells-1761188-supplementary.pdf]

Supplementary Data

Hypothalamic CREB regulates the expression of Pomc processing enzyme Pcsk2

Zanesco et al.

Supplementary Table S1. Macronutrient composition of the diets

Component	HFD	Standard Diet - SD
Sucrose	100	-
Dextrinized corn starch	132	-
Starch	199,5	550
Protein (casein 85%)	200	225
Soybean oil	40	45
Lard	228	-
Mineral mix	35	50
Vitamin mix	10	50
Fibers (cellulose)	50	80
L-Cystine	3	-
Choline bitartrate	2,5	-
**TOTAL (g)**	1000	1000

Supplementary Table S2. Key resources table

REAGENTE OR RESOURCE	SOURCE	IDENTIFIER
**Antibodies**		
Rabbit anti-pCREB	Cell Signaling	#9198
Rabbit anti-CREB	Cell Signaling	#4820
Donkey anti-rabbit IgG FITC	Abcam	#ab6798
Mouse anti-α-tubulina	Abcam	#ab7291
**Lentiviral Transduction Particles**		
shRNA-CREB	Sigma-Aldrich	TRCN0000301658
shRNA-CREB	Sigma-Aldrich	TRCN0000096633
shRNA-CREB	Sigma-Aldrich	TRCN0000096629
Scramble	Sigma-Aldrich	SHC002V
**Chemicals, Peptides, and Recombinant Proteins**		
Sodium palmiatate	Sigma-Aldrich	P9767
BSA fatty acid free	Roche Diagnostics Deutschland GmbH	10775835001
TRIzol Reagent	Thermo Fisher	Cat #15596018
High Fat Diet (HFD)	Pragsoluções Biociências	228 BAN
Chow diet	Nuvilab	CR-1
**Critical commercial assays**		
Rat/Mouse Neuropeptide Magnetic Bead Panel	Millipore	Cat. # RMNPMAG-83K
Taqman Gene Expression Master Mix	Applied Biosystems	Cat#4369016
**Experimental Models**		
Mouse: WT C57BL6 /J	Jackson Laboratory	Stock #664
POMC-Cre mouse	Jackson Laboratory	Stock #5965
B6.Cg-Gt(ROSA)26Sor$^{tm14(CAG-tdTomato)Hze}$/J	Jackson Laboratory	Stock #7914
**Oligonucleotides – primers gene**		
Pomc	Applied	Mm00435874_m1
Cart	Applied	Mm01811203_g1
Agrp	Applied	Mm00475829_g1
Npy	Applied	Mm01410146_m1
Pcsk1	Applied	Mm01345253_m1
Pcsk2	Applied	Mm00500981_m1
Prcp	Applied	Mm00804502_m1
Cpe	Applied	Mm00516341_m1
Il1β	Applied	Mm00434228_m1
Il6	Applied	Mm00446190_m1
Il10	Applied	Mm01288386_m1
Tgfβ1	IDT	138333258
Tlr4	Applied	Mm00445273_m1
Tnf	Applied	Mm00443258_m1
Nlrp3	Applied	Mm00840904_m1

Socs3	Applied	Mm00545913_s1
Gapdh	Applied	Mm99999915_g1
**Software**		
Excel	Microsoft	N/A
Word	Microsoft	N/A
PowerPoint	Microsoft	N/A
Prism 8.0	GraphPad Software	https://www.graphpad.com
Biorender	Biorender	N/A
Image J	National Institutes of Health	https://imagej.nih.gov/ij/

Supplementary Table S3. Quantitative analysis of western blot presented in Figure 3B.

Figure 3. B -Original western blotting membranes

		pCreb	α-Tubulin	
	**Sample**	**Average Pixel**	**Average Pixel**	**Ratio**
**Membrane 1**	**CT 1**	9.715.782	17.208.983	0,565
	**CT 2**	9.759.690	12.640.083	0,772
	**CT 3**	12.920.196	23.643.569	0,546
	**CT 4**	13.846.225	19.382.033	0,714
	**CT 5**	4.966.426	12.104.205	0,410
	1H 1	9.665.196	17.105.861	0,565
	1H 2	19.033.602	19.182.569	0,992
	1H 3	21.980.731	20.363.740	1,079
	1H 4	6.372.497	25.018.276	0,255
	1H 5	3.357.326	17.675.719	0,190
**Membrane 2**	3H 1	29.070.338	20.277.861	1,434
	3H 2	22.579.844	18.709.740	1,207
	3H 3	14.382.196	24.533.447	0,586
	3H 5	10.937.004	12.139.447	0,901
	6H 1	14.232.711	21.261.690	0,669
	6H 2	15.349.409	22.665.690	0,677
	6H 3	15.310.660	22.735.740	0,673
	6H 4	10.522.447	19.163.912	0,549
	6H 5	6.631.761	27.341.347	0,243
	9H 1	23.386.024	17.075.912	1,370
	9H 2	40.116.279	22.243.225	1,804
	9H 3	22.294.610	14.404.740	1,548
	9H 4	18.357.903	28.302.640	0,649
	9H 5	4.020.276	11.566.619	0,348

**pCREB/α-tubulin - Densitometry**					
	**SD**	**1H HFD**	**3H HFD**	**6H HFD**	**9H HFD**
50 uM	ratio	ratio	ratio	ratio	ratio
**Menbrane 1**	0,565	0,565	1,434	0,669	1,370
	0,772	0,992	1,207	0,677	1,804
**Mean**	0,67	0,78	1,32	0,67	1,59
**Menbrane 2**	0,546	1,079	0,586	0,673	1,548
	0,714	0,255	0,901	0,549	0,649
	0,410306	0,18994		0,242554	0,347576
**Mean**	0,56	0,51	0,74	0,49	0,85

**pCREB/α-tubulin - Densitometry**					
	**SD**	**1H HFD**	**3H HFD**	**6H HFD**	**9H HFD**
50 uM	% of control	% of control	% of control	% of control	% of control
**Menbrane 1**	84%	85%	214%	100%	205%
	116%	148%	181%	101%	270%
**Menbrane 2**	98%	194%	105%	121%	278%
	128%	46%	162%	99%	116%
	74%	34%	0%	44%	62%
**Mean**	**100%**	**117%**	**198%**	**101%**	**237%**
Error	0,06	0,19	0,23	0,08	0,26
DP	0,20	0,61	0,75	0,26	0,85

Supplementary Table S4. Quantitative analysis of western blot presented in Figure 3D.

Figure 3. D -Original western blotting membranes

		pCreb	α-Tubulin	
	Sample	Average Pixel	Average Pixel	Ratio
Membrane 1	Crtl 1	11.484.690	15.751.033	0,729
	Crtl 2	14.428.276	17.197.154	0,839
	Crtl 3	23.742.811	31.865.397	0,745
	Crtl 4	15.095.957	37.136.480	0,406
	Crtl 5	11.737.125	25.777.024	0,455
	3DHF1	8.819.619	14.454.861	0,610
	3DHF2	16.758.326	14.186.083	1,181
	3DHF3	34.043.099	31.683.773	1,074
	1WHF1	11.103.690	15.910.033	0,698
	1WHF2	9.085.033	16.117.447	0,564
	1WHF3	37.377.714	39.384.116	0,949
	1WHF4	15.946.489	24.023.560	0,664
	2WHF1	7.744.276	16.223.033	0,477
Membrane 2	2WHF2	11.958.569	20.857.205	0,573
	2WHF3	18.612.912	17.427.740	1,068
	2WHF4	8.924.418	26.164.752	0,341
	2WHF5	15.118.388	28.441.803	0,532
	4WHF1	7.366.740	26.022.397	0,283
	4WHF2	15.238.154	22.711.569	0,671
	4WHF3	20.896.640	21.903.740	0,954
	4WHF4	17.754.803	23.366.803	0,760
	4WHF5	5.852.953	13.821.581	0,423

pCREB/α-tubulin - Densitometry					
	SD	3D HFD	1W HFD	2W HFD	4W HFD
50 uM	ratio	ratio	ratio	ratio	ratio
Membrane 1	0,729139	0,610149	0,697905	0,477363	0,283092
	0,838992	1,181322	0,563677	0,573354	0,670942
	0,745097			1,068005	0,954022
**Mean**	0,771076	0,895735	0,630791	0,706241	0,636019
Menbrane 2	0,406	1,074	0,949	0,341	0,760
	0,455		0,664	0,532	0,423
**Mean**	0,43	1,07	0,81	0,44	0,59

pCREB/α-tubulin - Densitometry					
	SD	3D HFD	1W HFD	2W HFD	4W HFD
50 uM	% of control	% of control	% of control	% of control	% of control
Menbrane 1	95%	79%	91%	62%	37%
	109%	153%	73%	74%	87%
	97%			139%	124%
Menbrane 2	94%	249%	220%	79%	176%
	106%		154%	123%	98%
**Mean**	**100%**	**96%**	**90%**	**80%**	**104%**
Error	0,11	0,30	0,24	0,14	0,14
DP	0,38	0,95	0,79	0,45	0,46

Supplementary Table S5. Quantitative analysis of western blot presented in Figure 4B.

Figure 4. B -Original western blotting membranes

	pCreb	α-Tubulin	
Sample	Average Pixel	Average Pixel	Ratio
**SD1**	12.538.054	11.535.205	1,08694
**SD2**	7.420.811	28.224.083	0,26292
**SD3**	8.602.154	24.351.276	0,35325
**SD4**	15.229.326	23.071.690	0,66009
**SD5**	22.502.276	23.965.154	0,93896
**Média**			**0,66043**
**9H HFD1**	4.054.497	17.488.790	0,231834
**9H HFD2**	9.317.397	21.090.397	0,441784
**9H HFD3**	11.247.811	21.333.619	0,527234
**9H HFD4**	4.121.276	20.242.912	0,203591
**9H HFD5**	4.243.740	16.696.397	0,254171

pCREB/α-tubulin - Densitometry		
50 uM	SD	9H HFD
Membrane 1	165%	35%
	40%	67%
	53%	80%
	100%	31%
	142%	38%
**Mean**	**100%**	**50%**

Supplementary Table S6. Quantitative analysis of western blot presented in Figure 4G.

Figure 4. G -Original western blotting membranes

	pCreb	α-Tubulin	
Sample	Average Pixel	Average Pixel	Ratio
SD1	5.789.447	11.668.276	0,49617
SD2	8.344.740	15.123.205	0,55178
SD3	9.259.447	20.320.983	0,45566
SD4	12.089.811	18.486.912	0,65397
SD5	2.547.740	17.563.205	0,14506
**Mean**			**0,46053**
**4W HFD1**	9.713.276	14.775.569	0,657388
**4W HFD2**	18.058.983	14.352.790	1,258221
**4W HFD3**	6.888.740	19.155.497	0,359622
**4W HFD4**	10.929.033	17.515.619	0,623959
**4W HFD5**	6.626.861	26.917.397	0,246192

pCREB/α-tubulin - Densitometry		
50 uM	SD	4W HFD
Membrane 1	108%	143%
	120%	273%
	99%	78%
	142%	135%
	31%	53%
**Mean**	**100%**	**137%**

Supplementary Table S7. Quantitative analysis of western blot presented in Figure 5B.

Figure 5. B -Original western blotting membranes

	pCreb	α-Tubulin	
Sample	Average Pixel	Average Pixel	Ratio
Saline 1	10.323.983	17.085.154	0,604266
Saline 2	7.904.740	10.067.497	0,785174
Saline 3	7.664.983	14.193.154	0,540048
Saline 4	5.151.468	7.148.033	0,720683
**Mean**			**0,662543**
SP 2h 1	11.688.933	16.308.569	0,716736
SP 2h 2	12.963.397	16.685.154	0,776942
SP 2h 3	15.475.154	17.118.861	0,903983
SP 2h 4	15.538.317	14.273.740	1,088595
SP 2h 5	24.649.731	26.698.154	0,923275
SP 4h 1	5.501.740	9.281.033	0,592794
SP 4h 2	8.377.740	16.385.326	0,511295
SP 4h 3	6.827.175	9.969.326	0,684818
SP 4h 4	15.253.125	21.294.740	0,716286
SP 4h 5	22.178.539	29.193.447	0,759709

pCREB/α-tubulin - Densitometry			
50 uM	Saline	SP 2h	SP 4h
Membrane 1	91%	108%	89%
	119%	117%	77%
	82%	136%	103%
	109%	164%	108%
		139%	115%
**Mean**	**100%**	**133%**	**99%**

Supplementary Table S8. Quantitative analysis of western blot presented in Supplementary Figure S2a.

Figure Suppl 2. A -Original western blotting membranes								
	pCreb	α-Tubulin			Densitometria (%)			
**Sample**	**Average Pixel**	**Average Pixel**	**Ratio**		**Scramble**	shTRCN96629	shTRCN96633	shTRCN304358
Scramble 1	26079861,00	18071861,00	1,44		75%	94%	120%	36%
Scramble 2	26603104,00	13617497,00	1,95		101%	92%	68%	46%
Scramble 3	27338569,00	11379669,00	2,40		124%	86%	68%	22%
**Mean**			**1,93**	**Mean**	**100%**	**91%**	**85%**	**34%**
shTRCN96629	25775033,00	14217912,00	1,81					
shTRCN96629	20176740,00	11373426,00	1,77					
shTRCN96629	**17621326,00**	10547033,00	1,67					
shTRCN96633	25186154,00	10882912,00	2,31					
shTRCN96633	16094619,00	12295376,00	1,31					
shTRCN96633	21521861,00	16414255,00	1,31					
shTRCN304358	17215447,00	24807225,00	0,69					
shTRCN304358	22145518,00	25029690,00	0,88					
shTRCN304358	9652447,00	22946154,00	0,42					

1 Supplementary Figures

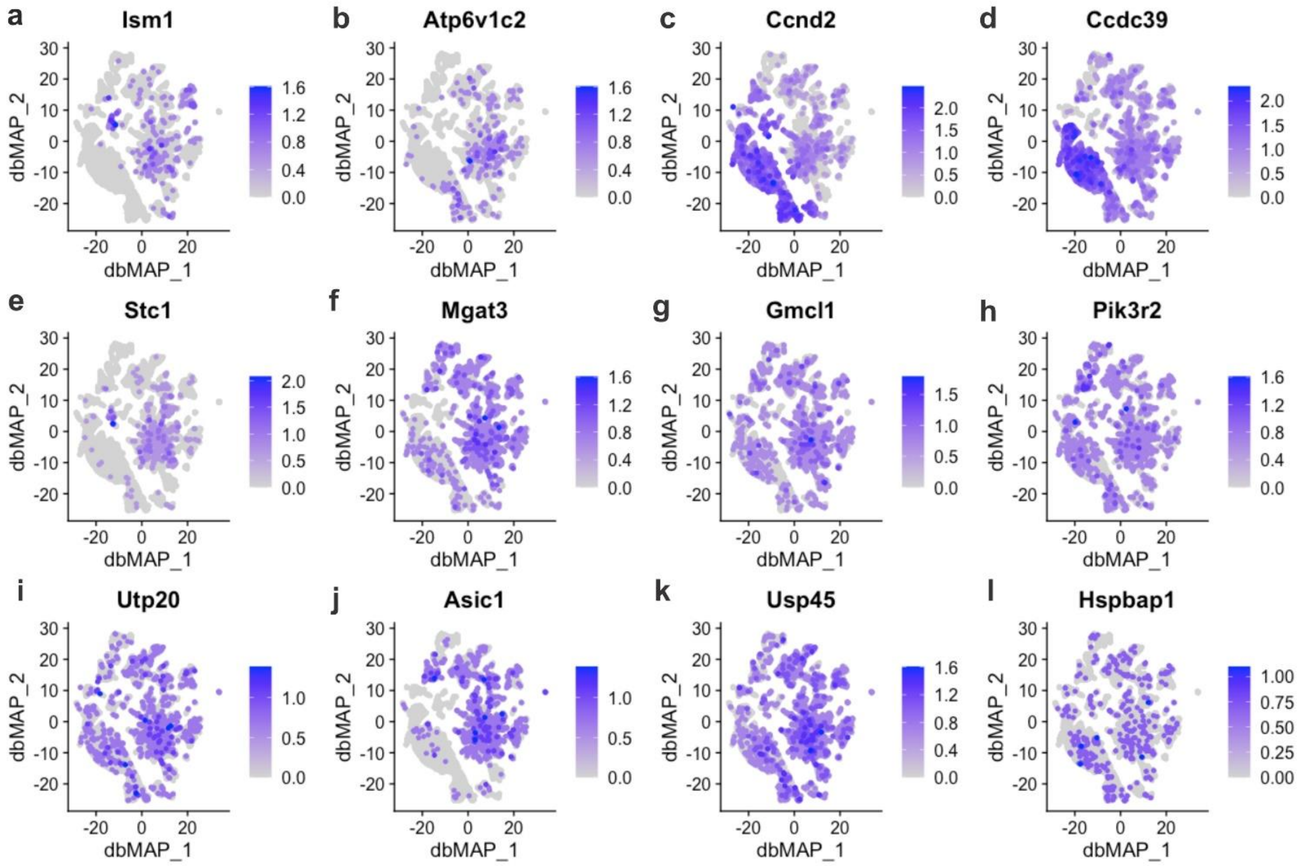

Supplementary Figure S1. Major Creb1 transcriptional targets in the Arc-ME. Gene expression of **(a)** *Ism1*; **(b)** *Atp6v1c2*; **(c)** *Ccnd2*; **(d)** *Ccdc39*; **(e)** *Stc1*; **(f)** *Mgat3*; **(g)** *Gmcl1*; **(h)** *Pik3r2*; **(i)** *Utp20*; **(j)** *Asic1*; **(k)** *Usp45* and **(l)** *Hspbap1*, the 12 major Creb1 transcriptional targets with higher relative importance (excluding unclassified/predicted genes).

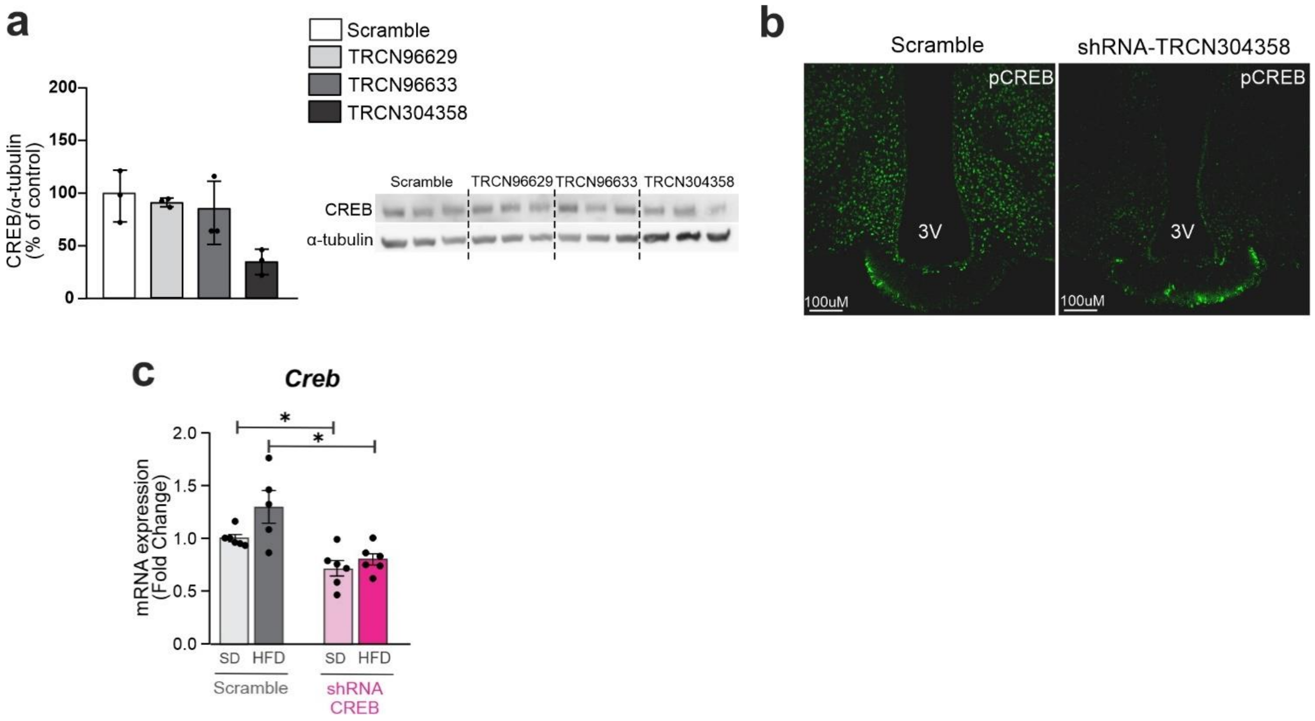

Supplementary Figure S2. Characterization of hypothalamic CREB knockdown. Adult male C57BL/6J mice were submitted to bilateral injection of lentivirus particles (Scramble and shRNA CREB) into the arcuate nucleus (ARC). Three distinct lentiviruses were tested for their efficiency to inhibit the expression of hypothalamic CREB. **(a)** Western blot of hypothalamic samples obtained 14 days after CREB down-regulation with different lentiviral-shRNA sequences; **(b)** Coronal brain section of mediobasal hypothalamus 14 days after the bilateral injection of shRNA CREB (TRCN304358) and Scramble particles into the ARC were immunostained with pCREB; the confocal microscope acquisition settings were kept the same for both groups. Hypothalamic mRNA expression of Creb in different conditions; **(c)** Adult male C57BL/6J mice were submitted a bilateral injection of lentivirus particles (Scramble or shRNA CREB) into the arcuate nucleus (ARC) and, after 10 days, were fed on SD or HFD for 3 days. Data are presented as means ± SEM. Two-way ANOVA followed by Sidak's post hoc test was used for statistical analyses. *p≤0,05 in comparison with groups indicated in the figure.

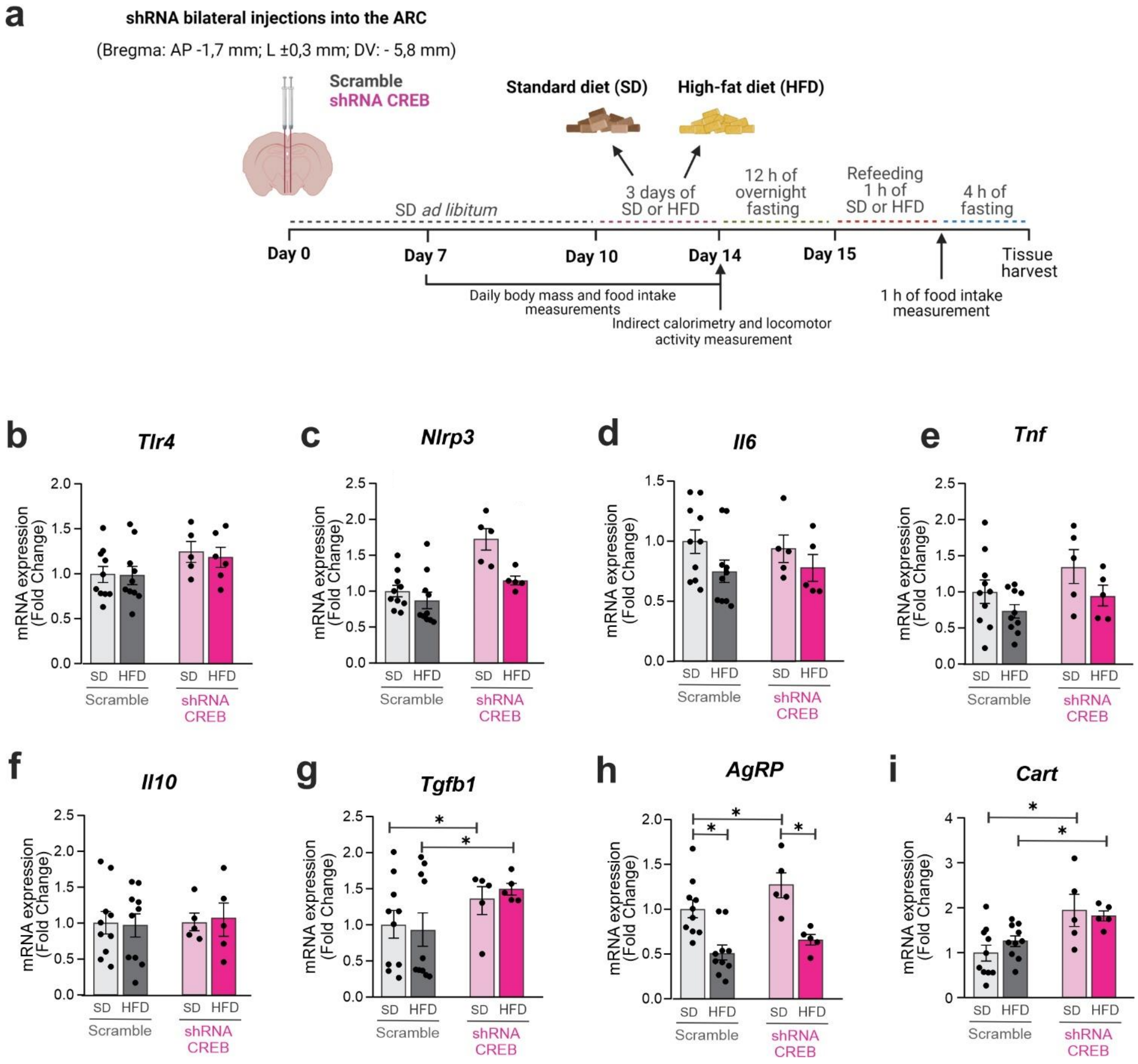

Supplementary Figure S3. Hypothalamic CREB down-regulation slightly modulates inflammatory markers and neuropeptides. Adult male C57BL/6J mice were submitted to bilateral injection of lentivirus particles (Scramble and shRNA CREB) into the arcuate nucleus (ARC) and, after 10 days, were fed on SD or HFD for 3 days. **(a)** Schematic representation of experimental protocol; mRNA gene expression of inflammatory **(b-g)** and neuropeptides **(h-i)**. Data are presented as means ± SEM. N= 5 - 15 mice in two different cohorts. Two-way ANOVA followed by Sidak's post hoc test was used for statistical analyses. *p≤0,05 in comparison with groups indicated in the figure.

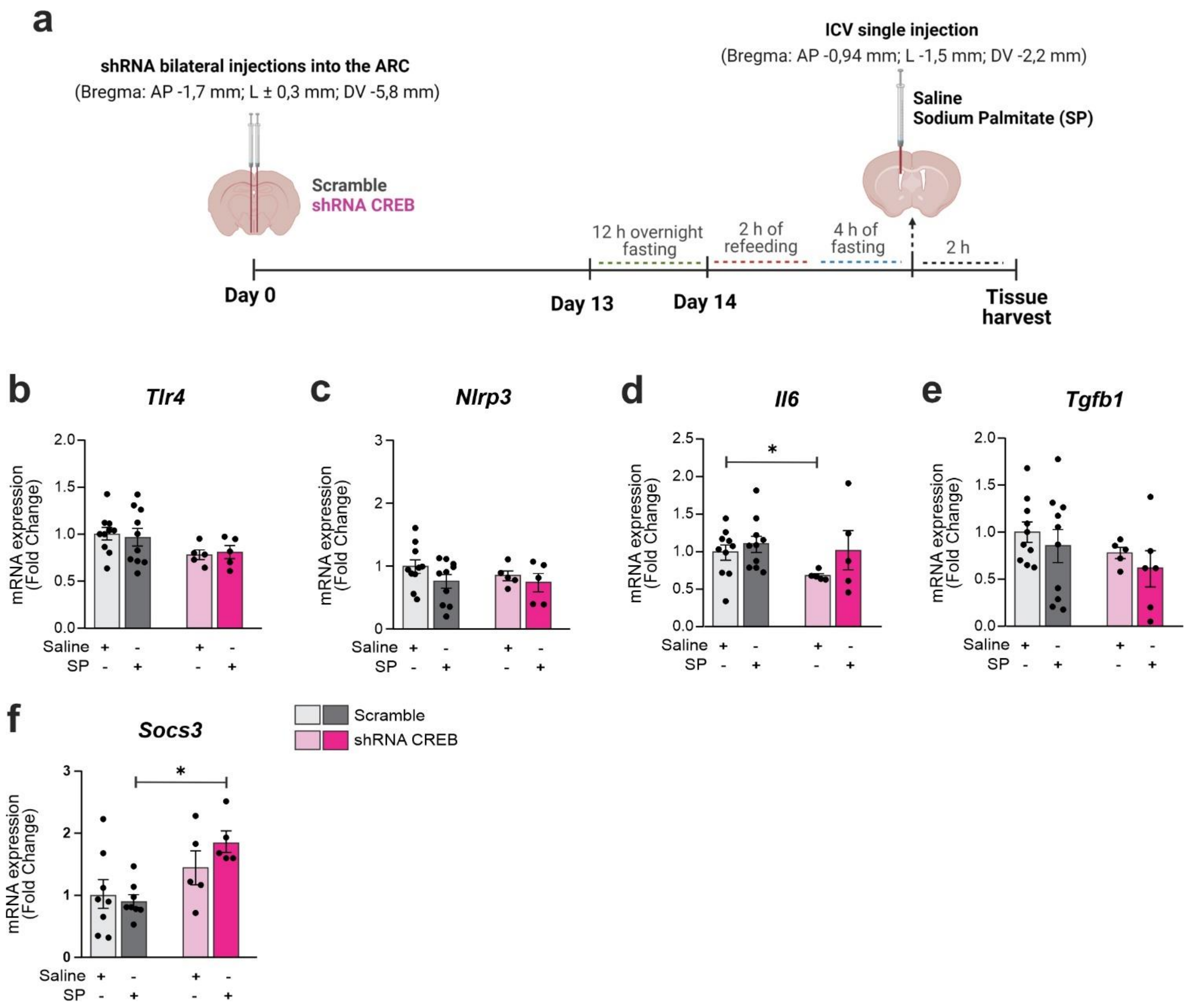

Supplementary Figure S4: Hypothalamic CREB knockdown does not modulate inflammatory markers upon palmitic acid stimulus. Adult male C57BL/6J mice were submitted a bilateral injection of lentivirus particles (Scramble or shRNA CREB) into the arcuate nucleus (ARC) and, after 14 days, were ICV injected with saline or sodium palmitate (SP) 30 µM. **(a)** Schematic representation of experimental protocol, hypothalami were harvested 2 hours after injection. **(b-f)** mRNA inflammatory markers. Data are presented as means ± SEM. N= 5-15 mice in two different cohorts. Two-way ANOVA followed by Sidak's post hoc test was used for statistical analyses. *p≤0,05 in comparison with groups indicated in the figure.
